# Grouping women of South Asian ethnicity for pregnancy research in New Zealand

**DOI:** 10.1111/ajo.13626

**Published:** 2022-10-25

**Authors:** Esti De Graaff, Lynn Sadler, Heena Lakhdhir, Rachel Simon‐Kumar, Roshini Peiris‐John, Wendy Burgess, Karaponi Okesene‐Gafa, Robin Cronin, Lesley Mccowan, Ngaire Anderson

**Affiliations:** ^1^ The University of Auckland Faculty of Medical and Health Sciences, Obstetrics & Gynaecology Auckland New Zealand; ^2^ Te Toka Tumai Auckland, Te Whatu Ora Health Auckland New Zealand; ^3^ Counties Manukau District, Division of Women's Health, Te Whatu Ora ‐ Health Auckland New Zealand; ^4^ The University of Auckland School of Population Health Auckland New Zealand; ^5^ The University of Auckland Section of Epidemiology and Biostatistics Auckland New Zealand

**Keywords:** ethnicity, risk factors, pregnancy complications, Asia, Western, New Zealand

## Abstract

**Background:**

The New Zealand (NZ) Ministry of Health ethnicity data protocols recommend that people of South Asian (SAsian) ethnicity, other than Indian, are combined with people of Japanese and Korean ethnicity at the most commonly used level of aggregation in health research (level two). This may not work well for perinatal studies, as it has long been observed that women of Indian ethnicity have higher rates of adverse pregnancy outcomes, such as perinatal death. It is possible that women of other SAsian ethnicities share this risk.

**Aims:**

This study was performed to identify appropriate groupings of women of SAsian ethnicity for perinatal research.

**Materials and Methods:**

National maternity and neonatal data, and singleton birth records between 2008 and 2017 were linked using the Statistics NZ Integrated Data Infrastructure. Socio‐demographic risk profiles and pregnancy outcomes were compared between 15 ethnic groups. Recommendations were made based on statistical analyses and cultural evaluation with members of the SAsian research community.

**Results:**

Similarities were observed between women of Indian, Fijian Indian, South African Indian, Sri Lankan, Bangladeshi and Pakistani ethnicities. A lower‐risk profile was seen among Japanese and Korean mothers. Risk profiles of women of combined Indian‐Māori, Indian‐Pacific and Indian‐New Zealand European ethnicity more closely represented their corresponding non‐Indian ethnicities.

**Conclusions:**

Based on these findings, we suggest a review of current NZ Ministry of Health ethnicity data protocols. We recommend that researchers understand the risk profiles of participants prior to aggregation of groups in research, to mitigate risks associated with masking differences.

## INTRODUCTION

Women of South Asian (SAsian) ethnicity in Western societies are at higher risk for adverse pregnancy outcomes compared with other ethnicities, including stillbirth, preterm delivery, and gestational diabetes mellitus (GDM).^1^, ^2^, ^3^, ^4^ Geographically South Asia includes Afghanistan, Bangladesh, Bhutan, India, the Maldives, Nepal, Pakistan, and Sri Lanka.^5^ Current international research combines women of SAsian ethnicities for analysis based on these definitions,^1^, ^6^, ^7^ even though ‘South Asian’ encompasses people of varied backgrounds. Historical heterogeneity of SAsian peoples and more recent migration patterns have resulted in socio‐cultural diversity both within South Asia, and in the rest of the world. The Indian diaspora is additionally the largest worldwide.^8^ Migrants from the Indian subcontinent made up for 3.4% of the total New Zealand (NZ) population at the time of the 2013 Census, increasing to 4.8% in 2018.^9^ Consecutively, while the total sum of births in NZ has declined over the last two decades, a growing number of births to women of Indian ethnicity has been observed each year.^10^ Considering the current birth‐trend, it has become increasingly important to understand and mitigate this risk of adverse pregnancy outcomes among women of SAsian ethnicity.

Some key characteristics of ethnicity recorded in NZ include that it is self‐defined, and that an individual may identify with more than one ethnic group. The use of ethnicity data in health research is addressed by the *Ethnicity Data Protocols for the Health and Disability Sector* by the Ministry of Health (MOH).^11^ According to this protocol, ethnicity data can be categorised at four different ‘levels’. In aggregation a reasonable level of detail is maintained for some ethnicities (such as Māori, Pacific Peoples, Chinese or Indian), while other minority groups are merged together despite large heterogeneity (such as other Asian ethnicities, African or Latin American). All SAsian groups, except Indian, are merged with Japanese and Korean ethnicity at level two aggregation, which is most commonly used in maternity research. If an individual identifies with multiple ethnicities, three forms of output are recommended: total response (ie a person is counted in each reported ethnic group), prioritised (ie a single ethnic group is allocated based on prioritisation tables outlined by the MOH), and sole/combination (ie categories of women with one or a combination of ethnic groups).^11^ Although ‘prioritised’ output of ethnicity data is often used in health research with the intention to fairly represent Māori, Pacific Peoples and ethnic minorities, while ensuring large enough groups for analyses, the appropriateness of this method has been questioned.^12^, ^13^

This study was performed to identify groupings of women of SAsian ethnicity for pregnancy research, with a comparison to current NZ ethnicity categorisation, as understanding the facilitators and barriers to health outcomes for ethnic minorities require populations with small numbers to be appropriately aggregated. Based on historical diaspora, we hypothesised that women of Indian, Sri Lankan, Bangladeshi and Pakistani ethnicity would be suitable for grouping.

## MATERIALS AND METHODS

This research was designed by a team comprising members of diverse ethnic and professional backgrounds relevant to this study. The study was approved by the University of Auckland Human Participants Ethics Committee, with reference number 024201. National maternity and neonatal data of singleton births between 2008 and 2017 were linked using the Statistics NZ Integrated Data Infrastructure (IDI).^14^ The following datasets were used: the Maternity Collection (MAT; including clinical data on all birth events from 20 weeks gestation); Births, Deaths and Marriages (BDM; infant and parent demographic data on all deliveries); the National Minimum Dataset (hospital discharge event data); the Mortality Collection (recording cause of death on all mortalities, including stillbirths); the Chronic Conditions dataset (records of individuals with one or more out of eight chronic conditions, such as diabetes); the 2013 and 2018 Census; immigration data (information on border movements, country of birth and visa type); and address notifications.

Individual ethnicity data was collected from BDM, the 2013 Census and MAT dataset; and used in that order depending on data availability. As women of SAsian ethnicity in NZ mostly identify with one ethnic group and rarely with more than two, the ‘sole/combination’ method was deemed most appropriate for this study. The following 15 ethnicities or combined ethnic groups were included: Indian, Fijian Indian, South African Indian, Sri Lankan, Bangladeshi, Pakistani, Afghan, Nepali, Indian‐Māori, Indian‐Pacific, Indian‐NZ European (NZE), Māori, Pacific Peoples, NZE, and Japanese and Korean (aggregated to Japanese/Korean). We identified a mother as Fijian Indian, when ethnicity was coded as ‘Fijian’ and ‘Indian’, ‘Fijian Indian’, or ‘Fijian Indian’ and ‘Indian’. Similarly mothers were classified as South African Indian, when ethnicity was coded as ‘South African’ and ‘Indian’. No births to women of Bhutanese or Maldivian ethnicities were found. Other combined groups were excluded from this study as these were either too small or too heterogenous.

### Statistical analyses

Analyses were performed using SAS 8.3 Enterprise Guide. Demographic characteristics were compared in univariate analyses using Kruskal‐Wallis and χ^2^ or Fisher's exact tests. Post hoc analyses were adjusted for multiple comparisons by applying the Dwass‐Steel‐Critchlow‐Fligner procedure or the Stepdown Bonferroni method. Investigated pregnancy outcomes included perinatal related mortality (deaths from 20 weeks gestation up to the 28th day after birth per 1000 total births), preterm birth (PTB; births <37 weeks gestation per 100 live births), caesarean section (CS; both elective and emergency procedures), assisted deliveries (AD; forceps and vacuum), induction of labour (IOL), hypertensive disorders of pregnancy (pregnancy‐induced hypertension, pre‐eclampsia and eclampsia)^15^ and gestational diabetes mellitus (GDM).^16^ If hypertensive disorders or GDM were not flagged, these were assumed ‘absent’. Births to women registered with a district health board (DHB; hospital providers of maternity care) were excluded from all analyses including body mass index (BMI), smoking, parity or trimester of booking. Analyses on CS, AD and IOL were based on nulliparous women only. Pregnancy outcome rates were compared by Mantel–Haenszel test and simple logistic regression methods. Odds ratio (OR) or adjusted odds ratio (aOR) estimates, and profile‐likelihood confidence intervals were computed. Women of Indian ethnicity, as the largest SAsian category, were used as the referent group in all analyses. No between‐group analyses were performed.

## RESULTS

**Figure 1:**
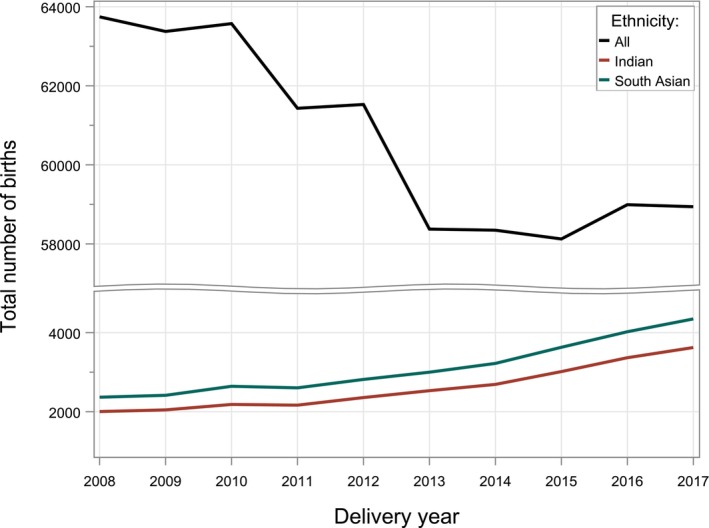
Total number of births by ethnicity, between 2008 and 2017 in New Zealand.

There were 606 435 singleton births in NZ between 2008 and 2017. An increasing number of births to women of SAsian ethnicity was observed over time, representing 3.7% of total births in 2008 and 7.4% in 2017 (Fig. 1). There were 31 074 births among mothers identifying as solely SAsian (Table 1). Ethnicity data was sourced from BDM for the majority of women (98.3%). Demographic characteristics of all individual groups are shown in Table 1. Pregnancy outcome rates are shown in Table 2. Some results were suppressed due to low counts, or secondarily suppressed to prohibit re‐calculation, following Statistics NZ guidelines.^17^

**Table 1:** Maternal and neonatal demographics by ethnicity

*N*		Indian	Fijian Indian	South African Indian	Sri Lankan	Bangladeshi	Pakistani	Afghan	Nepali	Indian‐Māori	Indian‐Pacific	Indian‐New Zealand European	Māori	Pacific	New Zealand European	Japanese/Korean
		25 989	1920	108	1767	396	1254	1344	324	165	147	351	67 467	47 844	265 599	5949
Maternal ethnicity data source (%)	BDM births	25 782 (99.2)	1881 (98.0)	108 (100.0)	1731 (98.0)	390 (98.5)	1242 (99.0)	1320 (98.2)	312 (96.3)	162 (98.2)	141 (95.9)	345 (98.3)	66 045 (97.9)	47 253 (98.8)	264 195 (99.5)	5913 (99.4)
	Census 2013	129 (0.5)	27 (1.4)	S	S	S	S	S	S	S	S	S	795 (1.2)	375 (0.8)	1137 (0.4)	S
	MAT	78 (0.3)	12 (0.6)	S	S	S	S	S	S	S	S	S	627 (0.9)	216 (0.5)	267 (0.1)	S
Maternal demographics																
Age (mean ± SD)	Years	29.5 (±4.4)	28.6 (±5.0)**	30.6 (±4.8)	31.6 (±4.4)**	29.2 (±5.0)	29.4 (±4.7)	27.2 (±5.3)**	29.0 (±4.2)	24.0 (±6.5)**	25.1 (±5.5)**	29.2 (±5.9)	26.1 (±6.4)**	28.0 (±6.4)**	30.3 (±5.8)**	33.1 (±4.4)**
BMI (mean ± SD)	kg/m^2^	24.4 (±4.5)	25.4 (±5.3)	23.9 (±4.5)	24.2 (±4.1)*	25.0 (±4.2)	24.5 (±4.3)	24.7 (±4.6)	24.3 (±3.9)*	27.0 (±6.0)	28.8 (±6.3)**	25.2 (±5.7)	28.6 (±6.3)**	32.4 (±6.8)**	25.6 (±5.4)**	21.4 (±3.0)**
BMI category (%)^†^	<25.0 kg/m^2^	12 531 (61.6)	750 (51.4)**	54 (62.1)	765 (63.6)	159 (55.2)	570 (63.1)	456 (57.6)	174 (62.4)	57 (40.4)**	39 (36.1)**	192 (60.4)*	18 540 (32.8)**	4167 (14.0)**	140 832 (56.3)**	4791 (89.3)**
	25–29.9 kg/m^2^	5724 (28.2)	438 (30.0)	24 (27.6)	336 (27.9)	99 (34.4)	228 (25.2)	237 (29.9)	87 (31.2)	45 (31.9)	27 (25.0)	75 (23.6)	17 739 (31.4)	7422 (24.9)	64 584 (25.8)	480 (8.9)
	≥30.0 kg/m^2^	2049 (10.1)	267 (18.3)	9 (10.3)	96 (8.0)	30 (10.4)	105 (11.6)	96 (12.1)	18 (6.5)	39 (27.7)	42 (38.9)	51 (16.0)	20 061 (35.5)	18 153 (60.8)	44 079 (17.6)	87 (1.6)
Smoking status (%)^†^	Yes	99 (0.5)	27 (1.9)**	S	S	S	S	S	S	54 (38.3)**	9 (8.3)**	27 (8.5)**	27 306 (48.3)**	3921 (13.1)**	25 866 (10.3)**	30 (0.6)
	No	20 229 (99.5)	1431 (98.1)	S	S	S	S	S	S	84 (59.6)	99 (91.7)	291 (91.5)	29 202 (51.6)	25 893 (86.8)	224 049 (89.6)	5337 (99.4)
Parity (%)^†^	0	11 499 (56.6)	789 (54.1)**	48 (55.2)	633 (52.6)*	147 (51.0)	327 (36.2)**	276 (34.8)**	153 (54.8)	63 (44.7)**	51 (47.2)**	162 (50.9)**	16 815 (29.7)**	8826 (29.6)**	104 113 (41.7)**	2727 (50.8)**
	1	7140 (35.1)	486 (33.3)	27 (31.0)	432 (35.9)	105 (36.5)	327 (36.2)	213 (26.9)	105 (37.6)	42 (29.8)	30 (27.8)	99 (31.1)	14 193 (25.1)	7362 (24.7)	91 059 (36.4)	1935 (36.1)
	≥2	1692 (8.3)	183 (12.6)	9 (10.3)	138 (11.5)	36 (12.5)	249 (27.6)	303 (38.3)	21 (7.5)	36 (25.5)	27 (25.0)	57 (17.9)	25 500 (45.1)	13 632 (45.7)	54 765 (21.9)	705 (13.1)
Trimester at booking (%)^†^	1st	12 735 (62.6)	810 (55.6)**	57 (65.5)	807 (67.1)	186 (64.6)	552 (61.1)	375 (47.3)**	174 (62.4)	69 (48.9)*	54 (50.0)	216 (67.9)	22 638 (40.0)**	9297 (31.2)**	187 503 (75.0)**	3822 (71.2)**
	2nd	6894 (33.9)	594 (40.7)	S	357 (29.7)	84 (29.2)	303 (33.6)	318 (40.2)	93 (33.3)	66 (46.8)	48 (44.4)	87 (27.4)	27 654 (48.9)	16 377 (54.9)	56 178 (22.5)	1293 (24.1)
	3rd/4th	702 (3.5)	54 (3.7)	S	39 (3.2)	18 (6.3)	48 (5.3)	99 (12.5)	12 (4.3)	6 (4.3)	6 (5.6)	15 (4.7)	6261 (11.1)	4158 (13.9)	6306 (2.5)	252 (4.7)
Lead maternity carer at registration (%)	DHB‐primary care	5637 (21.7)	459 (23.9)	21 (19.4)	561 (31.7)**	108 (27.3)*	351 (28.0)**	552 (41.1)**	45 (13.9)*	24 (14.5)	39 (26.5)	33 (9.4)**	10 857 (16.1)**	17 931 (37.5)**	15 468 (5.8)**	582 (9.8)**
	Non‐DHB care	20 331 (78.2)	1458 (75.9)	87 (80.6)	1203 (68.1)	288 (72.7)	903 (72.0)	792 (58.9)	279 (86.1)	141 (85.5)	108 (73.5)	318 (90.6)	56 559 (83.8)	29 841 (62.4)	249 996 (94.1)	5367 (90.2)
Deprivation index quintile (%)	Quintile 1	2409 (9.3)	162 (8.4)**	27 (25.0)**	261 (14.8)**	30 (7.6)*	126 (10.0)	105 (7.8)**	S	S**	S**	66 (18.8)**	1749 (2.6)**	942 (2.0)**	57 690 (21.7)**	1260 (21.2)**
	Quintile 2	3702 (14.2)	222 (11.6)	33 (30.6)	321 (18.2)	33 (8.3)	180 (14.4)	171 (12.7)	S	S	S	69 (19.7)	3666 (5.4)	1968 (4.1)	60 333 (22.7)	1479 (24.9)
	Quintile 3	5349 (20.6)	315 (16.4)	27 (25.0)	423 (23.9)	87 (22.0)	264 (21.1)	252 (18.8)	81 (25.0)	27 (16.4)	18 (12.2)	54 (15.4)	7056 (10.5)	3837 (8.0)	59 184 (22.3)	1485 (25.0)
	Quintile 4	7767 (29.9)	582 (30.3)	S	447 (25.3)	129 (32.6)	372 (29.7)	333 (24.8)	87 (26.9)	30 (18.2)	36 (24.5)	78 (22.2)	14 103 (20.9)	9204 (19.2)	52 116 (19.6)	1089 (18.3)
	Quintile 5	6402 (24.6)	606 (31.6)	S	294 (16.6)	111 (28.0)	300 (23.9)	474 (35.3)	87 (26.9)	78 (47.3)	78 (53.1)	75 (21.4)	39 990 (59.3)	31 224 (65.3)	33 699 (12.7)	588 (9.9)
Country of birth (%)	Missing	234 (0.9)	12 (0.6)	S	9 (0.5)**	S*	S**	18 (1.3)**	S*	S**	S**	S**	1839 (2.7)**	1236 (2.6)**	1068 (0.4)**	45 (0.8)**
	NZ born	1245 (4.8)	90 (4.7)	S	45 (2.5)	S	S	21 (1.6)	S	156 (94.5)	108 (73.5)	297 (84.6)	64 803 (96.1)	20 439 (42.7)	243 222 (91.6)	54 (0.9)
	Non‐NZ born	24 510 (94.3)	1818 (94.7)	S	1713 (96.9)	387 (97.7)	1233 (98.3)	1305 (97.1)	321 (99.1)	S	S	S	825 (1.2)	26 169 (54.7)	21 309 (8.0)	5850 (98.3)
Visa status (%)	Resident	17 523 (67.4)	1302 (67.8)*	93 (86.1)*	1191 (67.4)**	270 (68.2)*	816 (65.1)**	630 (46.9)**	177 (54.6)**							
	Student	351 (1.4)	S	S	S	S	S	S	S							
	Work	4527 (17.4)	264 (13.8)	6 (5.6)	288 (16.3)	87 (22.0)	285 (22.7)	207 (15.4)	105 (32.4)							
	Visitor	450 (1.7)	27 (1.4)	S	S	12 (3.0)	66 (5.3)	69 (5.1)	S							
	Refugee	9 (0.03)	S	S	42 (2.4)	S	S	297 (22.1)	24 (7.4)							
Religion (%)	No religion	621 (2.4)	51 (2.7)*	S**	36 (2.0)**	6 (1.5)**	15 (1.2)**	18 (1.3)**	9 (2.8)**	57 (34.5)**	18 (12.2)**	174 (49.6)**	27 990 (41.5)**	3171 (6.6)**	151 029 (56.9)**	2418 (40.6)**
	Buddhism	30 (0.1)	S	S	717 (40.6)	S	S	S	21 (6.5)	S	S	S	30 (0.04)	9 (0.02)	318 (0.1)	276 (4.6)
	Christianity	3558 (13.7)	216 (11.3)	36 (33.3)	378 (21.4)	6 (1.5)	42 (3.3)	18 (1.3)	24 (7.4)	30 (18.2)	72 (49.0)	84 (23.9)	16 572 (24.6)	33 933 (70.9)	71 583 (27.0)	1926 (32.4)
	Hinduism	11 139 (42.9)	801 (41.7)	45 (41.7)	330 (18.7)	18 (4.5)	S	S	225 (69.4)	9 (5.5)	9 (6.1)	6 (1.7)	30 (0.04)	42 (0.1)	84 (0.03)	S
	Islam	2451 (9.4)	582 (30.3)	12 (11.1)	84 (4.8)	297 (75.0)	987 (78.7)	1113 (82.2)	S	6 (3.6)	12 (8.2)	15 (4.3)	69 (0.1)	72 (0.2)	177 (0.1)	21 (0.4)
	Sikh	4287 (16.5)	S	S	S	S	S	S	S	S	S	S	9 (0.01)	18 (0.04)	9 (0.003)	S
	Other/multiple	1224 (4.7)	111 (5.8)	S	S	9 (2.3)	18 (1.4)	27 (2.0)	27 (8.3)	39 (23.6)	15 (10.2)	45 (12.8)	15 303 (22.7)	1962 (4.1)	28 638 (10.8)	561 (9.4)
Neonatal characteristics																
Gender (%)	Male	13275 (51.1)	957 (49.8)	57 (52.8)	900 (50.9)	213 (53.8)	630 (50.2)	675 (50.2)	159 (49.1)	84 (50.9)	72 (49.0)	177 (50.4)	34 818 (51.6)	24 561 (51.3)	136 098 (51.2)	2991 (50.3)
	Female	12 585 (48.4)	945 (49.2)	51 (47.2)	861 (48.7)	183 (46.2)	621 (49.5)	666 (49.6)	165 (50.9)	78 (47.3)	72 (49.0)	171 (48.7)	32 196 (47.7)	22 980 (48.0)	128 709 (48.5)	2952 (49.6)
Gestational age (mean ± *SD*)	Weeks	38.7 (±2.3)	38.5 (±2.4)	38.3 (±2.0)	38.6 (±2.1)*	38.5 (±2.1)	38.8 (±2.3)	39.1 (±2.0)**	39.1 (±1.7)	38.8 (±2.7)	39.0 (±1.5)	38.8 (±2.)	38.8 (±2.4)**	39.0 (±2.2)**	39.1 (±2.1)**	39.0 (±1.9)**
Birthweight (mean ± *SD*)	Grams	3123 (±571)	3130 (±598)	3038 (±540)	3181 (±555)*	3075 (±534)	3169 (±557)	3415 (±552)**	3218 (±513)	3222 (±665)	3355 (±482)**	3298 (±578)**	3319 (623)**	3567 (±634)**	3500 (±593)**	3278 (±504)**

(%)^†^Denominator: non‐DHB care Lead Maternity Carer at registration; S: suppressed value due to low count, or to prohibit re‐calculation; *P < 0.05, **P < 0.0001 compared with Indian ethnicity.

Abbreviations: BDM, births, deaths, and marriages; MAT, Maternity Collection; BMI, body mass index; DHB, district health board.

**Table 2:** Pregnancy outcomes by ethnicity

*N*		Indian	Fijian Indian	South African Indian	Sri Lankan	Bangladeshi	Pakistani	Afghan	Nepali	Indian‐Māori	Indian‐Pacific	Indian‐New Zealand European	Māori	Pacific	New Zealand European	Japanese/Korean
		25 989	1920	108	1767	396	1254	1344	324	165	147	351	67 467	47 844	265 599	5949
Obstetric complication																
Perinatal related mortality	Rate/1000 total births	13.9 (*n* = 25 989)	15.6 (*n* = 1920)	S	11.9 (*n* = 1767)	S	7.2 (*n* = 1254)	8.9 (*n* = 1344)	S	S	S	S	12.3 (*n* = 67 467)	12.9 (*n* = 47 844)	8.7 (*n* = 265 599)	6.6 (*n* = 5949)
	OR (95% CI)	Ref	1.13 (0.78–1.64)	S	0.86 (0.55–1.33)	S	0.51 (0.26–1.00)	0.64 (0.36–1.14)	S	S	S	S	0.89 (0.78–1.01)	0.93 (0.82–1.06)	0.62 (0.56–0.70)	0.47 (0.34–0.65)
Preterm birth	%	6.8 (*n* = 25 653)	7.8 (*n* = 1890)	11.4 (*n* = 105)	6.0 (*n* = 1755)	6.1 (*n* = 393)	5.5 (*n* = 1245)	4.5 (*n* = 1338)	3.7 (*n* = 324)	7.4 (*n* = 162)	S	8.7 (*n* = 345)	7.9 (*n* = 66 651)	5.7 (*n* = 47 223)	5.7 (*n* = 263 316)	4.5 (*n* = 5913)
	OR (95% CI)	Ref	1.16 (0.97–1.38)	1.78 (0.97–3.25)	0.88 (0.72–1.07)	0.90 (0.59–1.36)	0.81 (0.63–1.03)	0.65 (0.50–0.84)	0.53 (0.30–0.94)	1.10 (0.61–1.99)	S	1.31 (0.90–1.91)	1.18 (1.11–1.24)	0.83 (0.78–0.88)	0.82 (0.78–0.87)	0.64 (0.56–0.73)
Caesarean section	%	33.9 (*n* = 11 364)	33.8 (*n* = 780)	25.0 (*n* = 48)	44.0 (*n* = 627)	49.0 (*n* = 147)	31.5 (*n* = 324)	26.7 (*n* = 270)	41.2 (*n* = 153)	28.6 (*n* = 63)	29.4 (*n* = 51)	31.5 (*n* = 162)	18.7 (*n* = 16 290)	23.0 (*n* = 8631)	29.1 (*n* = 101 049)	28.8 (*n* = 2652)
	OR (95% CI)	Ref	1.00 (0.85–1.16)	0.65 (0.34–1.25)	1.53 (1.30–1.80)	1.87 (1.35–2.59)	0.90 (0.71–1.14)	0.71 (0.54–0.93)	1.36 (0.99–1.89)	0.78 (0.45–1.35)	0.81 (0.44–1.48)	0.90 (0.64–1.25)	0.45 (0.42–0.47)	0.58 (0.55–0.62)	0.80 (0.77–0.83)	0.79 (0.72–0.87)
Assisted vaginal delivery^†^	%	33.7 (*n* = 7476)	27.6 (*n* = 510)	25.0 (*n* = 36)	35.9 (*n* = 351)	33.3 (*n* = 72)	33.8 (*n* = 222)	37.3 (*n* = 201)	40.0 (*n* = 90)	21.4 (*n* = 42)	S	22.2 (*n* = 108)	11.9 (*n* = 13 188)	12.6 (*n* = 6612)	26.9 (*n* = 71 394)	30.6 (*n* = 1881)
	OR (95% CI)	Ref	0.75 (0.62–0.92)	0.66 (0.31–1.40)	1.10 (0.88–1.38)	0.99 (0.60–1.61)	1.01 (0.76–1.33)	1.17 (0.88–1.57)	1.31 (0.86–2.01)	0.54 (0.26–1.12)	S	0.56 (0.36–0.89)	0.27 (0.25–0.29)	0.28 (0.26–0.31)	0.72 (0.69–0.76)	0.87 (0.78–0.97)
Induction of labour	%	32.1 (*n* = 11 412)	35.6 (*n* = 783)	25.0 (*n* = 48)	30.1 (*n* = 627)	38.8 (*n* = 147)	29.6 (*n* = 324)	27.5 (*n* = 273)	30.0 (*n* = 150)	20.0 (*n* = 60)	23.5 (*n* = 51)	22.2 (*n* = 162)	20.3 (*n* = 16 344)	23.6 (*n* = 8688)	25.8 (*n* = 101 406)	25.1 (*n* = 2661)
	OR (95% CI)	Ref	1.17 (1.01–1.36)	0.70 (0.37–1.36)	0.91 (0.77–1.09)	1.34 (0.96–1.87)	0.89 (0.70–1.13)	0.80 (0.61–1.05)	0.91 (0.64–1.29)	0.53 (0.28–1.00)	0.65 (0.34–1.24)	0.60 (0.42–0.88)	0.54 (0.51–0.57)	0.65 (0.61–0.70)	0.74 (0.71–0.77)	0.71 (0.64–0.78)
Hypertensive disorders of pregnancy	%	4.9 (*n* = 25 989)	6.1 (*n* = 1920)	S	5.3 (*n* = 1767)	3.0 (*n* = 396)	3.1 (*n* = 1254)	3.1 (*n* = 1344)	3.7 (*n* = 324)	5.5 (*n* = 165)	4.1 (*n* = 147)	4.3 (*n* = 351)	4.6 (*n* = 67 467)	6.4 (*n* = 47 844)	4.6 (*n* = 265 599)	2.0 (*n* = 5949)
	aOR (95% CI)	Ref	1.22 (0.96–1.53)	S	1.26 (0.97–1.62)	0.77 (0.38–1.37)	0.74 (0.48–1.08)	0.84 (0.52–1.26)	0.81 (0.40–1.45)	1.49 (0.70–2.82)	1.27 (0.53–2.57)	0.88 (0.48–1.48)	1.12 (1.03–1.21)	1.06 (0.98–1.16)	1.00 (0.94–1.08)	0.48 (0.39–0.59)
Gestational diabetes	%	11.9 (*n* = 25 989)	15.3 (*n* = 1920)	11.1 (*n* = 108)	15.3 (*n* = 1767)	20.5 (*n* = 396)	12.2 (*n* = 1254)	8.3 (*n* = 1344)	11.1 (*n* = 324)	3.6 (*n* = 165)	6.1 (*n* = 147)	7.7 (*n* = 351)	2.7 (*n* = 67 467)	6.2 (*n* = 47 844)	2.7 (*n* = 265 599)	5.1 (*n* = 5949)
	aOR (95% CI)	Ref	1.18 (1.01–1.37)	0.82 (0.39–1.70)	1.26 (1.06–1.50)	1.74 (1.29–2.36)	0.94 (0.76–1.17)	0.64 (0.50–0.84)	0.94 (0.64–1.39)	0.19 (0.08–0.47)	0.26 (0.11–0.59)	0.46 (0.29–0.73)	0.11 (0.10–0.12)	0.18 (0.17–0.19)	0.17 (0.16–0.18)	0.58 (0.51–0.66)

Hypertensive disorders of pregnancy adjusted for maternal age, maternal body mass index, and parity; gestational diabetes adjusted for maternal body mass index.

^†^ Denominator: all vaginal births (excluding caesarean section); S: suppressed value due to low count, or to prohibit re‐calculation.

### Maternal characteristics

Significant differences in maternal characteristics such as maternal age, maternal BMI, smoking status and parity were observed between women of Indian and Afghan, Indian‐Māori, Indian‐Pacific, Indian‐NZE, and Japanese/Korean ethnicities. Outcomes observed among the combined ethnic groups were overall more similar to characteristics seen in women of their corresponding non‐Indian ethnic group. We identified that Afghan, Indian‐Māori, Indian‐Pacific, Māori and Pacific mothers generally booked later in pregnancy and more commonly resided in disadvantaged socio‐economic areas, compared to women of Indian ethnicity.

### Neonatal characteristics

Indian neonates were approximately 100–400 g lighter compared with Afghan, Indian‐Pacific, Indian‐NZE, Māori, Pacific, NZE and Japanese/Korean infants.

### Pregnancy outcomes

The perinatal related mortality rate (PRMR) was 13.9 for women of Indian ethnicity, which was similar to women of most other SAsian groups. Women of Japanese/Korean ethnicity had a significantly lower PRMR of 6.6 (OR 0.47, 95% CI 0.34–0.65). Compared with Indian women, PTB rates were reduced among Afghan (OR 0.65, 95% CI 0.50–0.84), Nepali (OR 0.53, 95% CI 0.30–0.94), and Japanese/Korean (OR 0.64, 95% CI 0.56–0.73) mothers. Women of Afghan, Japanese/Korean and all combined ethnicities had lower rates of CS, AD and IOL.

**Figure 2:**
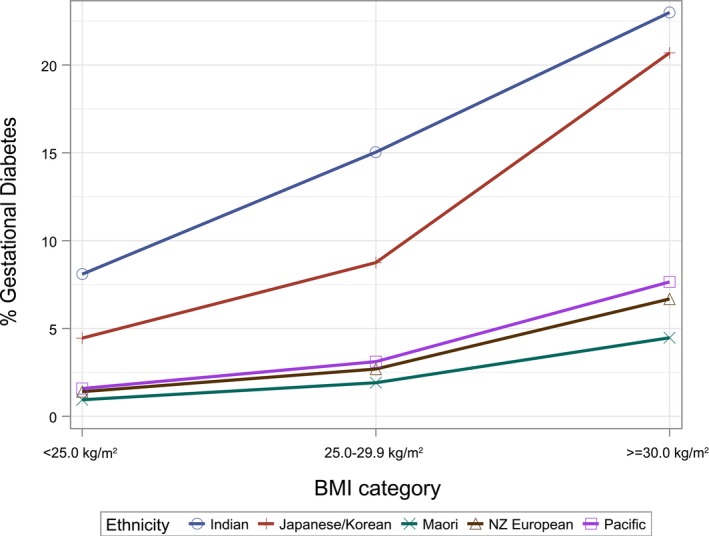
Gestational diabetes rates by body mass index category and ethnicity. Count (*N*) per body mass index category by ethnicity in Table 1.

Gestational diabetes rates varied between 11.1 and 20.5% among SAsian ethnicities, with the exception of Afghan mothers (8.3%, aOR 0.64, 95% CI 0.50–0.84). Mothers from the combined groups had significantly lower GDM rates, similar to their corresponding non‐Indian ethnicities, and the risk for Japanese/Korean mothers was approximately halved (aOR 0.58, 95%CI 0.51–0.66). As seen in Figure 2, women of Māori, Pacific and NZE ethnicities have lower GDM rates across all BMI categories, compared to Indian mothers.

## DISCUSSION

Many similarities in demographic characteristics and pregnancy complications were observed between women of Indian, Fijian Indian, South African Indian, Sri Lankan, Bangladeshi and Pakistani ethnicity. Therefore, we consider it appropriate to group these women for perinatal studies in the NZ setting. We acknowledge that some women of Fijian Indian ethnicity may identify more as Pacific than Indian due to historic events over the past 140 years.^18^ Although we observed a marginal shift in pregnancy risk factors between women of Indian and Fijian Indian ethnicities toward a Pacific risk profile, this was not associated with a significant alteration in pregnancy outcomes. For the purpose of pregnancy research in NZ, we thus propose to continue to aggregate women of Fijian Indian ethnicity with women from the Indian subcontinent, unless future research identifies that this trend has changed and there is a need for ethnicity data to be revised. In contrast, women of Afghan ethnicity seemed to differ in demographic characteristics and outcomes, with fewer adverse events. Afghan women also represent a different type of migrant, as they mostly belong to refugee groups in NZ, reflected by a larger proportion of women living in the most deprived neighbourhoods. Other factors which may influence pregnancy risk among refugees include educational background, health literacy, or length of residence in NZ. Finally, Nepali women have similar demographic characteristics to Indian mothers, with somewhat improved pregnancy outcomes. However, large cultural differences exist within this SAsian group.

Large differences were observed in almost all major pregnancy risk factors and outcomes between women of SAsian and Japanese/Korean ethnicities. The importance of correct aggregation by ethnicity, to best classify at‐risk mothers, is illustrated by the increased risk of GDM among SAsian mothers. As confirmed in earlier studies,^19^, ^20^ all SAsian groups in our cohort had increased rates of GDM compared to women of other ethnicities, even with significantly lower BMIs. With the current ethnicity prioritisation in health research, at‐risk women of Sri Lankan, Bangladeshi and Pakistani ethnicities would be grouped together with low‐risk mothers of Japanese/Korean ethnicity, masking the differences that exist.

A shift in risk profile was observed among the combined ethnic groups toward their second non‐Indian ethnicity. Boven and colleagues (2020) found similar results investigating the relationship between ethnicity and smoking rates using the ‘sole/combination’ output method and concluded that differences in important measures were undetectable by prioritised or total response output.^21^ In prioritised ethnicity output, women of Indian‐Māori or Indian‐Pacific ethnicities would be prioritised as either Māori or Pacific. Our findings support this method, as women of Māori and Pacific ethnicities are similarly overrepresented in certain pregnancy complications. However, this prioritised method does not accurately reflect the risk for women of Indian‐NZE ethnicity, as this lower‐risk combined group would be aggregated as Indian.

In order to better represent people from these SAsian groups in NZ, we therefore propose a revision of the current collection and aggregation methods of the MOH ethnicity data protocols, reflecting concerns raised during public consultation by Statistics NZ in 2019.^13^ An alternative grouping method, based on similar analyses as those performed in this study, could include aggregation by Central, South, South‐East, and East Asian ethnicities. Furthermore, although this study aims to group women with similar risk profiles, we acknowledge that a high level of diversity still exists between people of SAsian ethnicity. Current ethnicity data collection methods that record ‘Indian’ or ‘other Asian’ do not identify this internal diversity and therefore prohibit more nuanced discussion. An example of internal diversity includes religion, although a genetic study of SAsian people suggests that geographic location explains genetic variation better than religion, highlighting the importance of ancestry.^22^ It has been suggested to reconstruct classification based on people of north Indian and south Indian descent, emphasising this ancestral link.^23^ If the quality of ethnicity data collection in NZ were improved and included more detail, the accuracy of grouping could be increased further.

A strength of this study is the availability of high‐quality ethnicity data from various sources in the IDI. The recommendations presented are based on statistical analyses and cultural reflection, in consultation with researchers identifying with the relevant ethnic backgrounds. To our knowledge, no similar studies have been previously published. By performing this research, we were able to challenge commonly accepted protocols, with the aim to improve maternity research in NZ. This may ultimately lead to better understanding of risk and development of intervention strategies tailored to specific at‐risk groups.

There were some data limitations to this study. Exploratory analyses by level four ethnicity codes suggest low‐quality data collection for women of SAsian ethnicity. We suspect that Fijian Indian mothers were often incorrectly coded as ‘Indian’ and ‘Pacific’ separately, as in preliminary exploration of the dataset per year, the number of women coded as either Fijian Indian or two separate ethnicities were largely inversely correlated. This issue has been addressed by the ethnicity data protocols.^11^ In addition, even though NZ is known to have a growing population of South African Indians over the last decade,^24^ none were identified as such in our dataset. We expect similar inconsistencies may have occurred among other ethnic groups. Such incorrect coding may happen with inappropriate data collection. For example, women might identify as ‘Punjabi’, but are reported solely as ‘Indian’, and some healthcare professionals may be unaware that ‘Fijian Indian’ is acknowledged as a unique ethnicity. Furthermore, births to women registered with a DHB were excluded from all analyses including BMI, smoking, parity or booking trimester, due to a large amount of missing data. Anecdotally the variable ‘booking trimester’ may not accurately represent the timing of registration with a healthcare provider and it has not been validated. However, the MOH does use this variable in annual reports on clinical indicators for quality control.^25^ Additionally, ethnicities with poorer socio‐economic status generally booked later in pregnancy, as expected.^26^ Analyses on CS, AD and IOL were based on nulliparous women only, since we were not able to adjust for previous obstetric outcomes as an important risk factor.^27^ Further limitations were specific to the IDI. Missing BDM data on perinatal deaths, and a conservative linking method by Statistics NZ, restricted identification of clinical data in many perinatal death cases.^28^ In addition, data quality from the various sources is variable, with general inconsistencies in meta‐data, and no administrative data are available for those who did not access government services or do not reside in NZ.^29^

Confounding between ethnicity and ancestry (often corresponding to country of birth) is especially important when considering metabolic disorders such as GDM, where genetics or epigenetics may play a role. This study has shown that some risk factors can be culturally determined, as seen among the combined ethnic groups. In contrast, the high rate of GDM among Fijian Indian mothers (similar to Indian women) may indicate ancestral importance. Even though these intergenerational differences may explain some variance in pregnancy outcome,^2^ no analyses were performed between women of first‐ and second‐generation migrants in this study due to the relatively low number of SAsian women born in NZ. With increasing migration, ethnic groups will change over time, thus analyses should be repeated in future studies to continue correct grouping of individuals. As this study is specific to the NZ setting, we recommend other countries perform similar analyses within their unique population. While NZ‐based health research is generally conducted by ethnicity, surrogate variables are often used internationally, such as country of birth,^6^ nationality,^30^ race,^7^ or a combination.^31^ We argue that ethnicity together with ancestry captures the influence on health outcomes best, as beside a genetic component, major modifiable risk factors may be culturally determined.^32^

In conclusion, within the definition of ‘South Asian’, most sub‐groups can be combined for pregnancy research in NZ. However, as we identified some groups with differing socio‐demographic background and risk profiles, our data emphasises the need to justify aggregation by ethnicity. The importance of accurate and detailed ethnicity data collection is highlighted.

## DISCLAIMER

These results are not official statistics. They have been created for research purposes from the Integrated Data Infrastructure (IDI), which is carefully managed by Stats NZ. For more information about the IDI please visit https://www.stats.govt.nz/integrated‐data/.
